# Interaction of Biliverdin Chromophore with Near-Infrared Fluorescent Protein BphP1-FP Engineered from Bacterial Phytochrome

**DOI:** 10.3390/ijms18051009

**Published:** 2017-05-08

**Authors:** Olesya V. Stepanenko, Olga V. Stepanenko, Irina M. Kuznetsova, Daria M. Shcherbakova, Vladislav V. Verkhusha, Konstantin K. Turoverov

**Affiliations:** 1 Laboratory of Structural Dynamics, Stability and Folding of Proteins, Institute of Cytology, Russian Academy of Sciences, 4 Tikhoretsky ave., St. Petersburg 194064, Russian; lvs@incras.ru (O.V.S.); sov@incras.ru (O.V.S.); imk@incras.ru (I.M.K.); 2 Department of Biophysics, Peter the Great St. Petersburg Polytechnic University, 29 Polytechnicheskaya st., St. Petersburg 195251, Russian; 3 Department of Anatomy and Structural Biology, Albert Einstein College of Medicine, 1300 Morris Park ave., Bronx, NY 10461, USA; daria.shcherbakova@einstein.yu.edu; 4 Department of Biochemistry and Developmental Biology, Faculty of Medicine, University of Helsinki, 8 Haartmaninkatu st., Helsinki 00290, Finland

**Keywords:** bacterial phytochrome, iRFP, IFP, near-infrared fluorescent protein, GAF domain, biliverdin, competitive inhibition

## Abstract

Near-infrared (NIR) fluorescent proteins (FPs) designed from PAS (Per-ARNT-Sim repeats) and GAF (cGMP phosphodiesterase/adenylate cyclase/FhlA transcriptional activator) domains of bacterial phytochromes covalently bind biliverdin (BV) chromophore via one or two Cys residues. We studied BV interaction with a series of NIR FP variants derived from the recently reported BphP1-FP protein. The latter was engineered from a bacterial phytochrome RpBphP1, and has two reactive Cys residues (Cys15 in the PAS domain and Cys256 in the GAF domain), whereas its mutants contain single Cys residues either in the PAS domain or in the GAF domain, or no Cys residues. We characterized BphP1-FP and its mutants biochemically and spectroscopically in the absence and in the presence of denaturant. We found that all BphP1-FP variants are monomers. We revealed that spectral properties of the BphP1-FP variants containing either Cys15 or Cys256, or both, are determined by the covalently bound BV chromophore only. Consequently, this suggests an involvement of the inter-monomeric allosteric effects in the BV interaction with monomers in dimeric NIR FPs, such as iRFPs. Likely, insertion of the Cys15 residue, in addition to the Cys256 residue, in dimeric NIR FPs influences BV binding by promoting the BV chromophore covalent cross-linking to both PAS and GAF domains.

## 1. Introduction

The non-invasive imaging of biological processes in vivo is one of the most important problems of modern molecular and cell biology. The solution of this problem requires fluorescent proteins (FPs) with absorption and fluorescence spectra that correspond to the so-called “near-infrared (NIR) transparency window” of biological tissues where hemoglobin and melanin no longer absorb but water still does not absorb. Recently developed from bacterial phytochromes (BphPs) NIR FPs meet these requirements [1,2,3,4,5].

BphPs are involved in light regulation of motility and pigment synthesis [6,7,8,9,10]. To sense the light BphPs utilize as a chromophore a heme-derived linear tetrapyrrole, biliverdin IXα (BV) [11]. BphPs are reversibly switched by far-red and NIR illumination between two stable states that absorb at 680–710 nm (the Pr state) and 740–760 nm (the Pfr state). Canonical BphPs have the ground Pr state while BphPs termed as bathy phytochromes have the ground Pfr state [10,12,13,14,15]. BphPs are multi-domain proteins composed of a photosensory core module (PCM) and an output effector module. The PCM can be separated into the chromophore-binding domain (CBD), which is minimally required for chromophore binding and PHY (phytochrome-specific) domain, which is implicated in photoconversion of the chromophore between Pr and Pfr states and signal propagation to the effector module. The CBD part consists of the PAS (Per-ARNT-Sim repeats) domain and the GAF (cGMP phosphodiesterase/adenylate cyclase/FhlA transcriptional activator) domains. In the N-terminal extension of the PAS domain of BphPs there is the conservative Cys residue (Cys15), which binds the ligand through a thioether bond, while the GAF domain forms a pocket, which is able to hold BV [16,17]. The output effector modules of the most of BphPs are histidine kinases (HKs) [8,11,15]. Some BphPs possess a GGDEF/EAL domain [18,19] involved in second messenger signaling and the others possess a PAS/PAC domain involved in protein-protein interactions [10,12].

**Figure 1:**
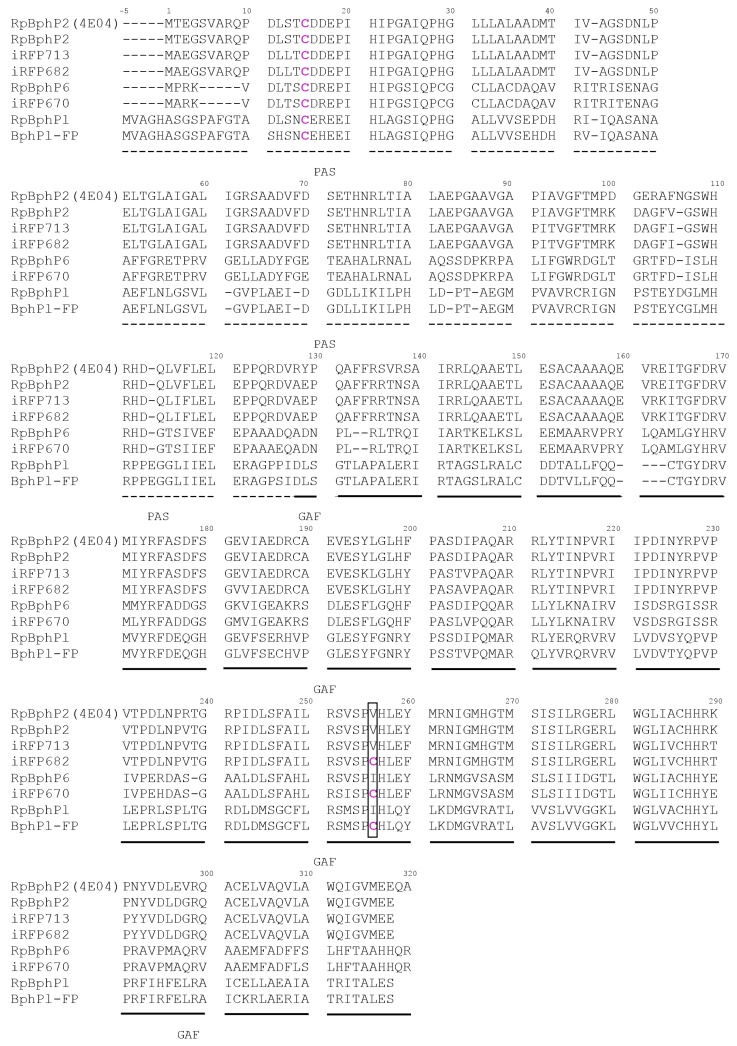
Alignment of amino acid sequences of the fluorescent protein BphP1-FP derived from bacterial phytochrome RpBphP1, near-infrared fluorescent protein iRFP670 and iRFP682/iRFP713 proteins with chromophore-binding domain (CBD) of their natural templates RpBphPl, RpBphP6 and RpBphP2 (and its crystallized variant, RpBphP2 (PBD ID: 4E04)), respectively. Conservative Cys residue in the Per-ARNT-Sim repeats (PAS) domain of all BphPs (Cys15) and engineered Cys residue in the cGMP phosphodiesterase/adenylate cyclase/FhlA transcriptional activator (GAF) domain of iRFP670, iRFP682 and BphPl-FP (Cys256) proteins are shown in magenta. PAS and GAF domains are highlighted with dashed and solid lines, respectively. Cys residue introduced into a conservative –SPXH- motif of the GAF domain is shown in the frame.

The domain organization of BphPs allowed developing from their truncated forms NIR FPs of different phenotypes. Permanently fluorescent NIR FPs [5,20,21,22,23,24] have been engineered on the basis of CBD of BphPs, photoactivatable NIR FPs [25]—on the basis of PCM of BphPs, and NIR reporters of protein-protein interaction [3,4,5]—on the basis of separate PAS and GAF domains of BphPs. Interestingly, the absorption/fluorescence spectra of NIR FPs of an Infra-red Fluorescent Protein (IFP) family (IFP1.4 [26], IFP2.0 [21], IFP1.4rev [22] and Wi-Phy [23]) engineered from *Deinococcus radiodurans* DrBphP differ no more than 16/14 nm, respectively. In contrast, among NIR FPs of a near-infraRed Fluorescent Protein (iRFP) family engineered from *Rhodopseudomonas palustris* RpBphP2 and RpBphP6 [20,24] the absorption/fluorescence spectral shift achieves 46/43 nm, respectively (iRFP670 vs. iRFP713). Recently reported novel NIR FP, termed BphP1-FP, engineered from *Rhodopseudomonas palustris* RpBphP1 also exhibits the blue-shifted absorbance and fluorescence (Table 1). It was shown that in BphP1-FP BV binds via a Cys256 residue (the amino acid numbering is given according to the alignment in Figure 1) introduced into a conservative -SPXH- motif of the GAF domain [27], resulting in two BV adducts. The blue-shift of these two BV based chromophores, both covalently bound to Cys256, originates from the smaller chromophore π-conjugated systems relative to a BV adduct bound to Cys15 in the PAS domain and found in natural BphPs and in common (red-shifted) NIR FPs, such as iRFP702, iRFP713 and iFRFP720 [27]. It has been proposed that the BV adducts with the smaller π-conjugated systems are formed in other blue-shifted NIR FPs, such as iRFP670 and iRFP682 [27,28]. Blue-shifted NIR FPs have a higher quantum yield than red-shifted NIR FPs, which can arise from two possibilities: either from the restricted motility of A ring of the BV-Cys256 adducts or from the decreased sensitivity of chromophore with shorter π-electron system to movements of A ring [29].

Insights into spectral characteristics of NIR FPs were obtained in studies of dimeric iRFPs and their mutants with Cys residues either in the PAS (Cys15) or in the GAF domains (Cys256), with Cys residues in both GAF and PAS domains, and lacking the Cys residues [28]. It has been found that the interaction of BV with dimeric NIR FPs is governed by inter-monomer and inter-domain allosteric effects. In dimeric iRFP variants without Cys15, a covalent binding of BV to Сys256 in one monomer allosterically inhibits the covalent binding of BV to another monomer whereas the presence of Cys15 allosterically promotes BV binding to Cys256 in both monomers [28].

In this paper, we performed spectral characterization of BphP1-FP and its mutants in buffered solutions and in the presence of denaturing agent, guanidine hydrochloride (GdnHCl). BphP1-FP contains the Cys residues (Cys15 and Cys256) in both the PAS and the GAF domains whereas its mutants have a Cys residue either in the PAS (BphP1-FP/C256I) or in the GAF domain (BphP1-FP/C15S), or no Cys residues (BphP1-FP/C15S/C256I). Absorbance and fluorescence measurements of these NIR FP variants revealed that the spectral characteristics of BphP1-FP and BphP1-FP/C15S are determined by BV covalently bound to Cys256 only and those of BphP1-FP/C256I—by BV covalently bound to Cys15 only. Our observations for monomeric variants of BphP1-FP confirm the conclusions made for dimeric iRFPs [28].

## 2. Results and Discussion

### 2.1. Properties of Fluorescent Protein BphP1-FP Engineered From Bacterial Phytochrome RpBphP1 and Its Mutants

**Figure 2:**
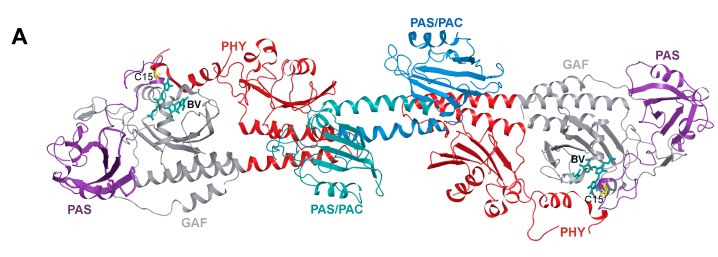

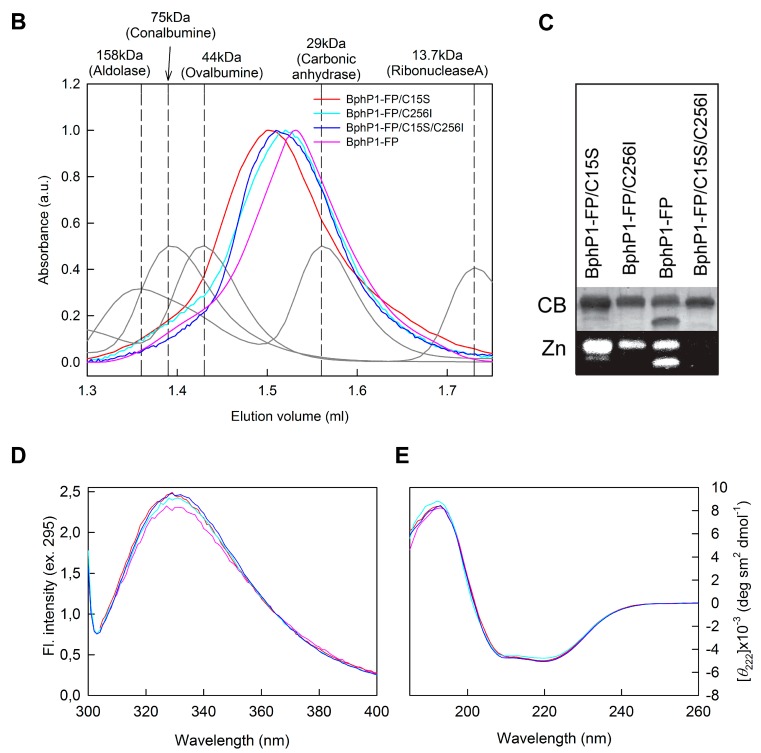
Characterization of BphPl-FP and its variants with different location of Cys residues. (**A**) Dimeric structure of RpBphP1-N70 without C-terminal HOS domain (PDB [31] ID: 4GW9 [30]). The graphic software VMD [32] and Raster3D [33] was used for drawing. Separate domains of RpBphP1-N70 monomer are differently colored; (**B**) Gel filtration of BphPl-FP, BphPl-FP/C15S, BphPl-FP/C256I and BphPl-FP/C15S/C256I proteins. The elution profiles of the proteins with known molecular mass used for the column calibration are shown in gray; (**C**) Covalent binding of biliverdin (BV) to proteins analyzed by sodium dodecyl sulfate polyacrylamide gel electrophoresis (SDS-PACE) followed by staining with Coomassie blue (CB) or by zinc-induced fluorescence of BV (Zn^2+^); (**D**) Tertiary structure of proteins tested using Trp fluorescence; (**E**) Secondary structure of BphPl-FP variants tested using far circular dichroism (CD) spectra.

Parental RpBphP1 from which a fluorescent protein BphP1-FP was engineered [27] is involved in the activation of expression of a cluster of more than 30 photosynthetic genes [10]. The output effector module of this protein is presented by the PAS/PAC domain and the HOS domain instead of HK [12]. Structure of RpBphP1-N70 (RpBphP1 without 10 kDa C-terminal part) [30] showed that its GAF domains do not participate in the dimer formation (Figure 2A), which is in contrast to RpBphP2 and RpBphP6 used as templates for dimeric iRFP series. The interface between the monomers of RpBphP1-N70 is composed by α-helical regions of about 30 amino acid residues from the PAS/PAC domains of each monomer and by several contacts between PAS/PAC domain of one monomer and PHY domain of another monomer [30]. Thus, RpBphP1-derived BphP1-FP, which lacks the PHY and effector domains, is possibly a monomer.

We have analyzed BphP1-FP and its mutants using gel filtration (Figure 2B). Elution profiles of BphP1-FP and its mutant contained a single peak with elution volume corresponding to the protein with molecular weight of about 35 kDa. These data confirmed that BphP1-FP and its mutant forms are monomers. Gel filtration experiments revealed the different degree of structure compaction of BphP1-FP variants. The value of elution volume of BphP1-FP/C15S and BphP1-FP/C15S/C256I indicated its slightly loosened structure as compared to BphP1-FP/C256I and BphP1-FP with the latter being the most compact (Figure 2B). Crystal structure of BphP1-FP/C15S showed that its N-terminal region was unstructured [27]. Apparently, the N-terminal region in this protein is not fixed because the BV chromophore in BphP1-FP/C15S is covalently attached to Cys256 residue of the GAF domain instead of Cys15 in the PAS domain. The same reason may contribute to the looser packing of BphP1-FP/C15S/C256I because the protein does not contain any Cys residues capable of BV binding. All BphP1-FP variants had almost identical circular dichroism (CD) spectra in the far-UV region and tryptophan fluorescence spectra (Figure 2D,E), which suggested the similarity in their secondary structures and in microenvironment of their single tryptophan residue, Trp281, located in the GAF domain. The zinc-staining assay confirmed the covalent binding of the BV chromophore in BphP1-FP, BphP1-FP/C256I, and BphP1-FP/S15S (Figure 2C). The sodium dodecyl sulfate polyacrylamide gel electrophoresis (SDS-PAGE) revealed two close bands for BphP1-FP containing both Cys15 and Cys256. In contrast, the SDS-PAGE of BphP1-FP variants containing Cys residue either in the PAS or in the GAF domain, or without Cys residues, showed single bands. Both bands of BphP1-FP exhibited Zn^2+^-induced fluorescence, which allowed us to attribute these bands to BphP-derived NIR FP with covalently bound BV. The same electrophoretic behavior was observed for other NIR FPs containing both Cys15 and Cys256 (e.g., iRFP670, iRFP682 and iRFP713/V256C [28]). On the other hand, the gel filtration on Superose 12 column of the dimeric NIR FP variants with two Cys residues and BphP1-FP studied here showed the single elution peak. This data indicate that BphP1-FP and other NIR FPs with both Cys15 and Cys256 residues are heterogeneous in the denatured state on SDS-PAGE but homogenous in the native state. It was shown recently that about a half of protein molecules of miRFP670 (this is a NIR FP recently designed from BphP1-FP [5]) contains a BV chromophore simultaneously bound via thioether bonds to both Cys20 and Cys253 residues [34] (they correspond to Cys15 and Cys256 according to the numering in current work). The CBD domain of bacterial photoreceptors contains a figure-of-eight knot with four crossing of polypeptide chain. Thus the crosslinking of the polypeptide chain via BV chromophore and both Cys residues would prevent protein from untying in denatured state. The topologically trapped knotted structure would be more compact and would show higher electrophoretic mobility. It can be assumed that second band on SDS-PAGE of BphP1-FP and dimeric NIR FP variants with both Cys15 and Cys256 residues arises from doubly-bounded BV chromophore, similarly to that found in miRFP670, which is a progeny of BphP1-FP.

We previously demonstrated that in dimeric NIR FPs [28], containing Cys residues in PAS or GAF domain only, inter-monomer allosteric inhibition of the covalent attachment may occur. These NIR FP variants have the covalently bound BV only in one monomer of the protein. For the NIR FPs with Cys256 residues in GAF domains only the fraction of non-covalently bound BV was seen as a red-shifted shoulder in the absorption and fluorescence spectra. The dimeric NIR FPs with Cys15 residues in PAS domains only demonstrated complex two-step denaturation behavior with non-covalently bound BV dissociating at lower denaturant concentration as compared to covalently attached to Cys15 BV. In the NIR FPs with two cysteine residues in both GAF and PAS domains the allosteric inhibition of covalent BV binding is overcome and covalent BV attachment to the Cys256 of both GAF domains yields a narrow blue-shifted absorption and fluorescence spectra. The absence of allosteric inhibition of covalent BV binding in the NIR FP variants with cysteine residues in both GAF and PAS domains allowed assuming the inter-domain allosteric influence of Cys15 in PAS domains to the structure of GAF domains.

We hypothesized that study of monomeric NIR FPs should give additional information about the BV interaction with BphPs and inter-domain and inter-monomer allosteric effects. Therefore, we further characterized BphP1-FP and its mutants using absorption spectroscopy and NIR fluorescence.

**Figure 3:**
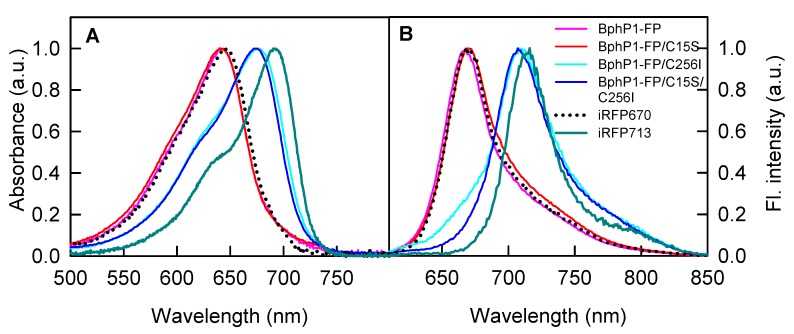
Spectral properties of BphPl-FP and its variants with different location of Cys residues. Absorbance (**A**) and fluorescence (**B**) spectra of BphPl-FP and its mutants are shown. Absorbance and fluorescence intensity (Fl. intensity) are presented in arbitrary units (a.u.). Fluorescence emission was excited at 600 nm.

BphP1-FP/C256I variant had a red-shifted absorption and fluorescence spectra, which are typical for NIR FPs with BV covalently bounded to Cys15 (Figure 3, Table 1). The fluorescence spectrum of BphP1-FP/C256I contained a small shoulder at the short-wavelength region, which may reflect the binding of protoporphyrin IX by the protein [35]. Protoporphyrin IX impurities may also explain the low quantum yield of BphP1-FP/C256I (Table 1). Absorption and fluorescence spectra of BphP1-FP/C15S/C256I mutant without Cys15 and Cys256 residues practically coincided with the spectra of BphP1-FP/C256I. As it was shown previously, despite the different chemical structure of non-covalently associated BV molecule and BV adduct covalently bound to Cys15, the both chromophores have equally extended π-conjugated electronic systems [27,28].

The absorption and fluorescence spectra of BphP1-FP/C15S and BphP1-FP proteins were almost identical and blue-shifted relative to the corresponding spectra of BphP1-FP/C256I variant by 34 and 38 nm, respectively (Figure 3, Table 1). BphP1-FP/C15S and BphP1-FP had narrow absorption and fluorescence spectra and almost the same spectral characteristics (Table 1). Because the absorption and fluorescence spectra of BphP1-FP/C15S did not contain red-shifted shoulder and spectral characteristics of this protein were similar to that of BphP1-FP, we concluded that there was no non-covalently bound BV in BphP1-FP/C15S. The spectral properties of BphP1-FP and BphP1-FP/C15S were, thus, determined only by covalently bound BV. The presence of two BV adducts covalently bound to Cys256, via either С3^1^ atom or С3^2^ atom of the A ring, was found in the crystal structure of BphP1-FP/C15S [27]. However, these BV adducts were spectrally indistinguishable and had similar fluorescence lifetimes [29], which did not allow discriminating between them. BphP1-FP probably contains two BV adducts also as closely related miRFP670 was shown to contain equimolar content of BV covalently bound to Cys256 via С3^2^ atom of the A ring and BV covalently bound to Cys256 via С3^1^ atom of the A ring and to Cys15 via С3^2^ atom of the A ring [34]. Despite these two BV chromophores are chemically diverse, their π-electronic system is identical leading to similar spectral profile of the chromophores [34].

### 2.2. Properties of BphP1-FP and Its Mutants in Denaturant

Spectral characteristics of BphP1-FP/C256I in the buffer solution do not show whether the protein contains non-covalently bound BV due to spectral identity of a free BV molecule in solution and a BV adduct that is covalently bound to Cys15. Therefore, we used an approach developed in for dimeric NIR FPs, which allows discriminating the chromophore forms differently assembled with a protein [28]. This technique consists in a comparison of the denaturant concentrations at which a BV chromophore dissociates from different mutants. Dissociation of a non-covalently bound BV chromophore occurs at the lower denaturant concentrations than those at which the covalently bound BV adduct dissociates. This is reflected in the complex two-step dependency of the protein absorbance at a specific wavelength on the denaturant concentration recorded for some variants of dimeric iRFPs [28].

**Figure 4:**
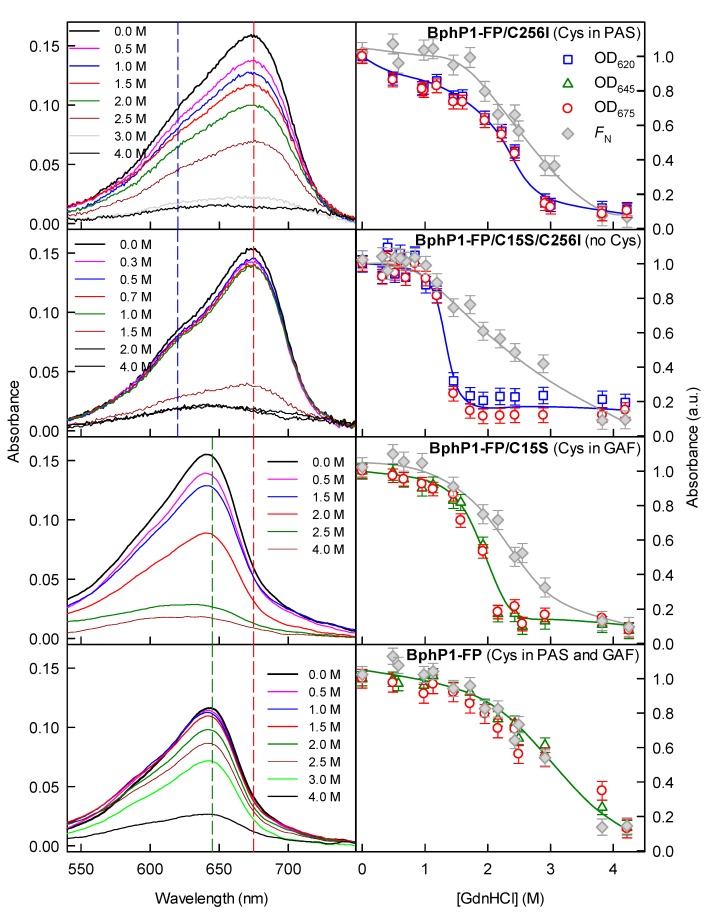
GdnHCl-induced changes in absorption of BphPl-FP variants. Absorption spectra are shown in the left panels. The numbers on the curves indicate the final denaturant concentrations in the protein samples. In the right panels the dependences of optical density at selected wavelengths (620 and 675 nm (blue and red vertical dashed lines in the left panels), or at 645 and 675 nm (green and red vertical dashed lines in the left panels)) on the GdnHCl concentration are presented. The data were normalized to BphPl-FP absorption at the respective wavelength in buffered solution. Stability of the protein structure against GdnHCl-induced unfolding is shown as the dependences of the part of native molecules *F*_N_ on the GdnHCl concentration (gray symbols). *F*_N_ was calculated from ellipticity at 222 nm. The selection of the wavelengths in these experiments was based on the observation that Cys256-BV adduct has the blue-shifted absorption with respect to Cys15-BV adduct and non-covalently bound BV. Thus, in the case of NIR FP proteins with Cys256 in GAF domains only we used wavelengths corresponding to the absorption maxima of Cys256-BV and non-covalently bound BV, 645 and 675 nm for BphP1-FP variants. In the NIR FPs proteins with Cys15 in PAS domains only, covalently and non-covalently bound chromophores are spectrally indistinguishable. In this case additionally to the maximum of Cys15-BV absorption (675 nm), the wavelength at blue-edge of protein absorption spectra (i.e., 620 nm) was selected as control.

We applied this approach to BphP1-FP and its mutants. Figure 4 shows the GdnHCl induced changes in the spectral properties of BphP1-FP variants over a broad range of denaturant concentrations. These data demonstrated that BphP1-FP/C256I did not contain non-covalently bound BV chromophore, since the absorbance dependences in the fluorescence maximum and at red-edge of the absorption spectrum changed simultaneously. The results have confirmed that BphP1-FP/C15S and BphP1-FP variants also contained the single (or spectrally indistinguishable) spectral form of the BV chromophore. The absence of non-covalently bound BV in monomeric NIR FPs confirms that in dimeric NIR FPs the binding of the BV chromophore is influenced by inter-monomer interaction. Our results corroborate with previous findings and indicate that the GAF/GAF domain interaction in BphPs is functionally important. In this regard, it has been shown that the structural changes in photosensory module of *Deinococcus radiodurans* DrBphP in the photoactivated Pfr:Pfr state [36] is identical to those observed in equmolar Pfr:Pr state of the DrBphP dimer [37]. That means that in dimeiric BphPs, the first BphP monomer can sense a light absorption by the second monomer. It has been also shown that a monomeric variant of the photosensory module of DrBphP can acquire the light-induced structural changes but with a reduced rate relative to that of the dimeric DrBphP [38]. Moreover, the disruption of the GAF/GAF interaction in the photosensory module and DrBphP was fatal for a thermal dark reversion from the Pfr photoactivated state to the Pr ground state [39].

**Figure 5:**
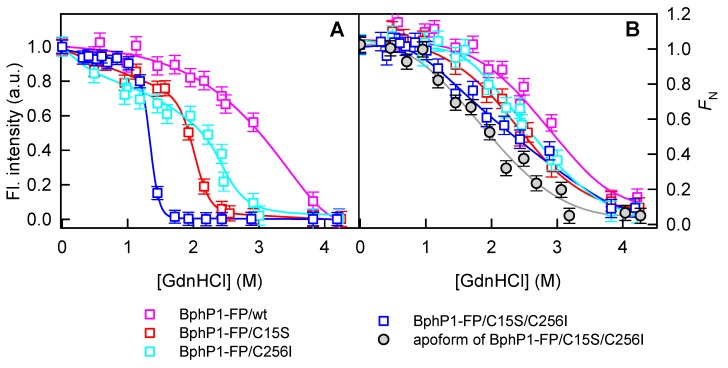
Stability of BphPl-FP and its variants in the holoform state against GdnHCl denaturation. (**A**) Changes in chromophore fluorescence intensity corrected to the total absorbance of the solution at the excitation wavelength, as described in “Materials and methods” section, are shown. Fluorescence was excited at 645 nm and recorded at 670 nm for BphPl-FP (pink squares) and BphPl-FP/C15S (red squares). Fluorescence was excited at 675 nm and recorded at 710 nm for BphPl-FP/C256I (cyan squares) and BphPl-FP/C15S/C256I (blue squares); (**B**) Dependences of the part of native molecules *F*_N_ on the GdnHCl concentration calculated from ellipticity at 222 nm. Data for BphPl-FP/C15S/C256I in apoform are also shown (solid gray circles).

BphP1-FP showed the most stable structure as compared to BphP1-FP/C15S, as well as to other variants (Figure 5). Dimeric NIR FPs with the Cys residues in both PAS and GAF domains were also characterized by the increased protein stability relative to their single-Cys variants [28,40]. We previously assumed that the Cys residue at position 15 affects the protein structure. Currently we can propose that the change of the structure is induced by double linking of BV to PAS and to GAF domains as that observed for miRFP670 [34]. This agrees well with gel filtration data. Indeed, BphP1-FP had even slightly more compact structure than BphP1-FP/C256I (Figure 2B) wherein the N-terminal region is restricted by a covalent bond of BV with Cys15. Thus enhanced stability and increased compactness of BphP1-FP was related to the fraction of protein cross-linked via BV chromophore. For miRFP670, protein, which is derived from BphP1-FP [34], a mechanism of the BV chromophore formation via subsequent binding of BV to Cys15 and then to Cys256 was suggested. Probably the covalent binding of BV to Cys15 in PAS domain stabilizes the chromophore in the conformation appropriate for the formation of covalent linkage with Cys256 in GAF domain. Based on this we can propose that in dimeric NIR FPs the same inter-domain interaction facilitates the covalent binding of BV in the second monomer of dimer. Thus, dimeric NIR FPs with introduced Cys15 residue in addition to the Cys256 residue, possibly, contain BV-Cys256 adduct in one monomer and Cys15-BV-Cys256 adduct in the second monomer. The later can explain the increased stability of dimeric NIR FPs with the Cys residues in both PAS and GAF domains. Since BV-Cys256 and Cys15-BV-Cys256 adducts are spectrally indistinguishable, the dimeric NIR FPs with the Cys residues in both PAS and GAF domains do not show spectral heterogeneity.

## 3. Materials and Methods

### 3.1. Plasmids, Mutagenesis, Protein Expression and Purification

The BphP1-FP DNA was amplified and cloned into a pBAD/His-B vector (Invitrogen, Carlsbad, CA, USA) using BglII and EcoRI sites. LMG194 host cells (Invitrogen, Carlsbad, CA, USA) were co-transformed by pWA23h plasmid for the expression of heme oxygenase under the rhamnose promoter [20] and pBAD/His-B plasmid encoding BphP1-FP and its variants with polyhistidine tags on the N-termini. Bacterial cells were grown in RM medium (1X M9 salts, 2% casamino acids, 1 mM MgCl2, 1 mM thiamine) supplemented with ampicillin and kanamycin. The expression of heme oxygenase was initiated first by 0.02% rhamnose. After incubation of cell culture for 5 h at 37 °C the expression of the target protein was induced by 0.002% arabinose followed by the incubation of cell culture for 12 h at 37 °C and for 24 h at 18 °C. Proteins were purified with affinity chromatography on a Ni-nitrilotriacetic acid (NTA) agarose column (GE Healthcare, Chicago, IL, USA). The Ni-NTA elution buffer contained 100 mM EDTA instead of imidazole. The exchange of elution buffer to phosphate buffered saline (PBS) (50 mM NaH_2_PO_4_, 150 mM NaCl, pH 8.0) was made with PD-10 desalting columns (GE Healthcare, Chicago, IL, USA). The final purification was achieved with ion-exchange chromatography on a MonoQ column (GE Healthcare, Chicago, IL, USA). The apoform of BphPl-FP/C15S/C256I was expressed in LMG194 cells. The overnight LMG194 culture was grown for 2–3 h at 37 °C; then protein synthesis was induced by 0.002% arabinose. The subsequent steps of expression and purification of protein in apoform were the same as for proteins in holoform.

The purity of the proteins was tested by sodium dodecyl sulfate polyacrylamide gel electrophoresis (SDS-PAGE) in a 12% polyacrylamide gels [41]. The protein was concentrated and stored in 20 mM Tris/HCl buffer, pH 8.0. The absorbance of the protein samples did not exceed 0.2, and the measurements were performed in 20 mM Tris/HCl buffer, pH 8.0, containing 1 mM tris (2-carboxyethyl) phosphine (TCEP).

### 3.2. Spectral and Biochemical Characterization of Purified Proteins

Absorption experiments were performed using a U-3900H spectrophotometer (Hitachi, Tokyo, Japan) with microcells 101.016-QS 5 mm × 5 mm (Hellma, Müllheim, Germany) at room temperature. The fluorescence spectra were recorded using a Cary Eclipse spectrofluorometer with 10 × 10 cells (Agilent Technologies, Mulgrave, Australia).

The extinction coefficient was determined by comparing the absorbance values at the far-red peak with the absorbance value at the Soret peak at about 390 nm. The extinction coefficient of tested proteins at the Soret band was supposed to be equal to that of free BV (39,900 M^−1^·cm^−1^). Nile blue dye was used as a standard for the evaluation of quantum yield. The chromophore binding with BphP1-FP and its variants was assayed by zinc-induced fluorescence and staining with Coomassie Blue of protein samples separated by SDS-PAGE [42].

Gel filtration experiments on BphP1-FP and its variants were performed on a Superose 12 PC 3.2/30 column (GE Healthcare, Chicago, IL, USA) using an AKTApurifier system (GE Healthcare). The samples of BphP1-FP and its variants were prepared in buffer consisting of 50 mM NaH_2_PO_4_, 150 mM NaCl, pH 8.0. Then 10 µL of protein sample was loaded on the column equilibrated with the same buffer. A set of molecular weight chromatography standards (GE Healthcare) were used for column calibration.

### 3.3. Protein Unfolding Assay

The protein unfolding was initiated by mixing 50 µL of the native protein with 500 µL of a buffer solution containing the desired concentration of GdnHCl (Sigma-Aldrich, St. Louis, MO, USA). The concentration of the stock GdnHCl solution was determined by the refraction coefficient. The dependences of the chromophore absorbance, fluorescence and ellipticity at 222 nm on the GdnHCl concentration for the BphP1-FP and its variants were recorded at 23 °C after protein incubation in a solution of an appropriate denaturant concentration at 23 °С for 24 h. Further increases in the equilibration time did not result in noticeable changes in the detected characteristics. The recorded fluorescence intensity was corrected for the primary inner filter effect according to the approach in [43,44].

### 3.4. Circular Dichroism Measurements

Jasco-810 spectropolarimeter (Jasco, Tokyo, Japan) was used for the measurement of CD spectra in the far UV range (from 260 to 190 nm). Three scans of CD spectra was collected, averaged and corrected by buffer solution background for every probe.

### 3.5. Fitting of Denaturation Curves

A two-state model was applied for fitting of the equilibrium dependences of the ellipticity at 222 nm on the GdnHCl concentration [45]:

(1) $$S=\frac{S_{N}+S_{U}K_{N-U}}{1+K_{N-U}}$$

(2) $$K_{N-U}=\exp\left(\frac{-\Delta G_{N-U}^{0}+m_{N-U}[D]}{RT}\right)$$

(3) $$K_{N-U}=F_{U}/F_{N}=(1-F_{N})/F_{N}$$

taking into account

(4) $$S_{N}=a_{N}+b_{N}[D]$$

(5) $$S_{U}=a_{U}+b_{U}[D]$$

where *S* is the ellipticity at 222 nm at the measured GdnHCl concentration; [*D*] is the guanidine concentration; *m* is the linear dependence of $\Delta G_{N-U}$ on the denaturant concentration; $\Delta G_{N-U}^{0}$ is the free energy of unfolding at 0 M denaturant; *F_N_* and *F_U_* are the fractions of native and unfolded molecules, respectively; and *S_N_* and *S_U_* are the signal of the native and unfolded states, respectively; a*_N_*, b*_N_*, a*_U_* and b*_U_* are constants. Fitting was made in Sigma Plot software (Systat Software Inc., San Jose, CA, USA).

## 4. Conclusions

**Figure 6:**
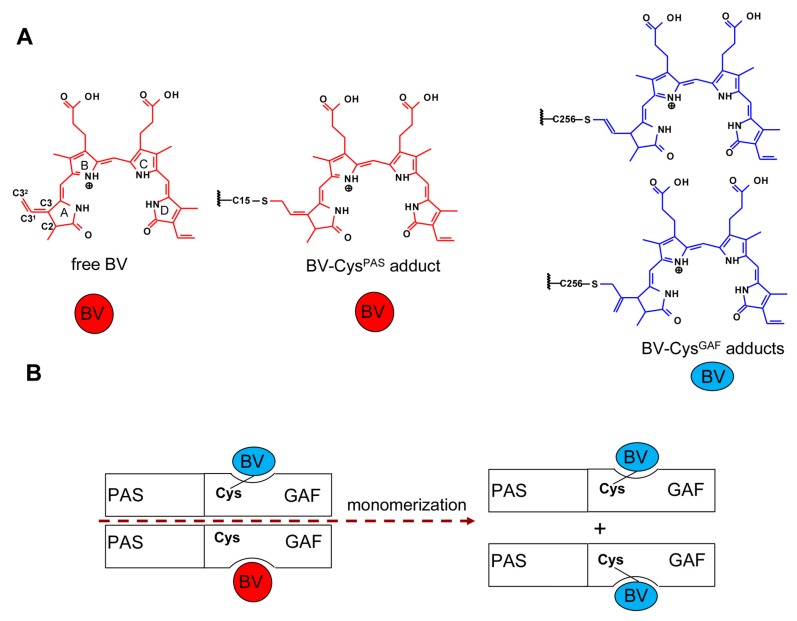
Scheme of the inter-monomeric effects in dimeric and monomeric NIR FPs. (**A**) Chemical structures of BV chromophores; (**B**) In dimeric NIR FPs inter-monomeric allosteric influence on the BV interaction with the protein results in covalent binding of BV only with one monomer of dimmer (the case of NIR FPs with Cys ^GAF^ only is shown). Monomeric BphP1-FP variants contained only covalently bound chromophore.

We have studied the biochemical, structural and spectral properties of BphP1-FP protein and its mutants in buffer solutions and in the presence of denaturant. We have found that in contrast to the iRFP family of NIR FPs, all BphP1-FP variants are monomers. The BphP1-FP variants containing either a Cys15 residue or a Cys256 residue, or both cysteine residues, bound the BV chromophore covalently only. The absence of the non-covalently bound BV in the monomeric BphP1-FP variants indicates the substantial inter-monomeric allosteric influence on the BV interaction with the protein monomers in dimeric NIR FPs (Figure 6). The inter-domain interaction, most likely via cross-linking of PAS and GAF domains to BV chromophore, contributes to the stability of monomeric NIR FPs. As the result, the BphP1-FP protein having two cysteine residues, Cys15 and Cys256, is spectrally identical to the BphP1-FP mutants with a single Cys256 residue but exhibits the notably higher protein stability. The similar structural changes in dimeric NIR FPs eliminate inhibition of BV covalent binding of and increase their stability.

## Figures and Tables

**Table 1:** Spectral and biochemical properties of fluorescent protein BphP1-FP, engineered from bacterial phytochrome RpBphP1, and its variants with different location of Cys residues.

NIR FP	Absorbance Maximum (nm)	FWHM ^1^ of Absorption Spectrum (nm)	Emission Maximum (nm)	FWHM of Emission Spectrum (nm)	Extinction Coefficient (M^−1^·cm^−1^)	Quantum Yield (%)	Molecular Brightness ^2^ vs. iRFP713 (%)
iRFP713 ^3^	692	66	713	38	98,000	6.3	100
iRFP670 ^3^	644	78	670	42	110,000	12.2	217
BphP1-FP	643	77	668	42	66,800	13.8	149
BphP1-FP/C15S	643	80	670	46	71,100	12.1	139
BphP1-FP/C256I	677	89	708	54	63,400	3.8	39
BphP1-FP/C15S/C256I	675	85	707	50	69,200	4.6	52

^1^ FWHM, full width at half maximum of spectrum; ^2^ Molecular brightness is a product of extinction coefficient and quantum yield. ^3^ Dimeric near-infrared fluorescent proteins iRFP713 and iRFP670.
